# Early Risk Stratification in Non-Classical Congenital Adrenal Hyperplasia Based on Newborn 17-OHP Screening Values, Hormonal Findings, and Genotype

**DOI:** 10.3390/jcm15072631

**Published:** 2026-03-30

**Authors:** Jessica Munarin, Gerdi Tuli, Enza Pavanello, Luisa De Sanctis

**Affiliations:** 1 Department of Pediatric Endocrinology, Regina Margherita Children’s Hospital, AOU Città della Salute e della Scienza, 10124 Turin, Italy; jessica.munarin@unito.it (J.M.); luisa.desanctis@unito.it (L.D.S.); 2 Department of Pediatrics, University of Turin, 10124 Turin, Italy; 3 Department of Laboratory Medicine, Regina Margherita Children’s Hospital, AOU Città della Salute e della Scienza, 10124 Turin, Italy; epavanello@cittadellasalute.to.it

**Keywords:** newborn screening, non-classical congenital adrenal hyperplasia, 17-hydroxyprogesterone

## Abstract

**Background/Objectives**: Non-classical congenital adrenal hyperplasia (NCCAH) due to 21-hydroxylase deficiency represents the mildest form of congenital adrenal hyperplasia and is frequently diagnosed only after the onset of clinical signs in childhood. Newborn screening programs for CAH are primarily designed to detect classical forms and show limited sensitivity for NCCAH. The clinical significance of neonatal 17-hydroxyprogesterone (17-OHP) values below recall thresholds remains incompletely defined. **Methods**: We retrospectively analyzed clinical, auxological, hormonal, and genetic data from pediatric patients diagnosed with NCCAH between 2018 and 2023 at a tertiary referral center. Neonatal screening 17-OHP concentrations, basal and ACTH-stimulated 17-OHP levels at diagnosis, bone age advancement, pubertal status, and hydrocortisone treatment were evaluated. Correlations between hormonal parameters, age at onset, and treatment dose were assessed. **Results**: Thirty-five patients (30 females) were included, with a mean age at clinical onset of 7.52 ± 0.36 years for females and 6.25 ± 0.29 years for males. Premature pubarche was the most frequent presenting sign (94.3%), and central precocious puberty was diagnosed in 31.4% of cases. The mean neonatal screening 17-OHP level was 4.53 ± 0.7 ng/mL; only two patients exceeded the screening recall cut-off. At diagnosis, mean basal and ACTH-stimulated 17-OHP levels were 15.1 ± 3.35 and 55.2 ± 11.3 ng/mL, respectively. Age at clinical onset was inversely correlated with both basal and stimulated 17-OHP levels, while hydrocortisone dose correlated positively with biochemical severity. Bone age advancement was observed in all patients. **Conclusions**: Most children with NCCAH display mildly elevated neonatal 17-OHP values that do not trigger screening recall. Higher biochemical severity is associated with earlier clinical presentation and higher glucocorticoid requirements. Neonatal 17-OHP concentrations, even when below cut-off values, may represent an early indicator of disease severity and warrant further investigation.

## 1. Introduction

Congenital adrenal hyperplasia (CAH) encompasses a group of autosomal recessive disorders caused by defects in adrenal steroid biosynthesis, leading to impaired cortisol production and compensatory hypersecretion of adrenocorticotropic hormone (ACTH). Among the various enzymatic defects, 21-hydroxylase deficiency accounts for more than 95% of cases and is associated with a wide clinical spectrum, ranging from severe neonatal forms to milder late-onset variants [1,2].

Late-onset or non-classical congenital adrenal hyperplasia (NCCAH) represents the mildest phenotype and is characterized by partial 21-hydroxylase deficiency [3]. NCCAH is considerably more prevalent than classical CAH, with an estimated prevalence of approximately 1:1000 in the general population, although significant variability has been reported according to ethnicity and genetic background [4,5,6,7,8]. The clinical heterogeneity of NCCAH largely reflects residual enzyme activity, which is determined by specific *CYP21A2* genotypes and compound heterozygous mutation patterns [4,5,6]. In contrast to classical congenital adrenal hyperplasia (CAH), in which lifelong glucocorticoid replacement is essential for survival, treatment in non-classical CAH (NCCAH) is directed primarily toward controlling progressive hyperandrogenic symptoms rather than preventing adrenal insufficiency. Most individuals with NCCAH maintain normal or near-normal intra-adrenal cortisol secretion, and ACTH levels are typically not elevated, reflecting the milder degree of 21-hydroxylase impairment. Nevertheless, the extent to which cortisol production is truly preserved remains debated. Although a modest reduction in enzyme activity (approximately 20–50%) is generally considered sufficient to sustain adequate cortisol output, several studies have reported suboptimal cortisol responses to standard-dose ACTH stimulation in a substantial proportion of patients, with 30–60% demonstrating peak cortisol values below conventional sufficiency thresholds [9,10,11,12,13,14]. These findings have prompted some authors to recommend glucocorticoid therapy even in asymptomatic individuals, particularly when nonspecific symptoms improve with treatment. However, the clinical significance and long-term implications of these biochemical abnormalities remain uncertain, and management decisions—especially for patients without overt symptoms—continue to be a matter of ongoing controversy.

In childhood, NCCAH typically presents with signs of androgen excess manifesting as precocious or early puberty, including premature pubarche, accelerated linear growth, advanced bone age, acne, and, less frequently, virilization or menstrual irregularities during adolescence [4,5]. Prolonged exposure to elevated adrenal androgens may also promote early activation of the hypothalamic–pituitary–gonadal axis, resulting in central precocious puberty (CPP), which can further accelerate skeletal maturation and negatively impact adult height outcome [15,16,17,18,19,20].

Newborn screening programs for CAH, based on the measurement of 17-hydroxyprogesterone (17-OHP) in dried blood spots, have been widely implemented and have markedly reduced morbidity and mortality associated with classical salt-wasting forms of the disease [21,22,23,24]. However, these screening strategies were specifically designed to detect severe phenotypes and show limited sensitivity for detecting NCCAH [23,24,25,26,27].

Several factors contribute to the low detection rate of NCCAH through neonatal screening. Affected individuals typically display only mildly elevated 17-OHP concentrations at birth, reflecting sufficient residual enzyme activity to prevent neonatal adrenal insufficiency [23]. In addition, screening cut-off values are intentionally set to minimize false-positive results, particularly in preterm or stressed neonates, which further limits the ability to detect mild forms of the disease [23]. Consequently, most patients with NCCAH have neonatal 17-OHP levels above the population mean but below the threshold for recall, resulting in false-negative screening outcomes [23].

Nevertheless, accumulating evidence suggests that neonatal 17-OHP concentrations in patients later diagnosed with NCCAH are frequently higher than those observed in the general population and may represent an early biochemical signature of partial 21-hydroxylase deficiency [22,23]. This observation supports the concept of a continuous relationship between neonatal hormonal patterns and subsequent clinical expression, rather than a strict dichotomy between classical and non-classical CAH. However, the clinical relevance of these mildly elevated neonatal values and their association with disease severity, age at onset, and treatment requirements remain incompletely defined, as potential overlap may also exist between NCCAH patients and newborns without NCCAH.

In the absence of reliable early diagnostic markers, NCCAH is frequently diagnosed only after the onset of clinical signs during childhood, often when significant bone age advancement has already occurred [7,19]. Confirmation of the diagnosis relies on ACTH stimulation test and subsequent genetic analysis of the *CYP21A2* gene [28,29].

Radioimmunoassay (RIA) has historically been used as a confirmatory test because it offers greater specificity than first-tier immunoassays by reducing cross-reactivity with fetal adrenal steroids. Although RIA improves the positive predictive value of CAH screening, its performance remains suboptimal. Recent comparative analyses demonstrate that RIA still yields a substantial proportion of false positives and is outperformed by more advanced chromatographic techniques. For example, LC-MS/MS confirmatory testing achieves a positive predictive value of 49% compared with 30% for RIA, underscoring the limitations of RIA in distinguishing true CAH from physiological neonatal steroid elevations [30].

Liquid chromatography-based assays, particularly liquid chromatography–tandem mass spectrometry (LC-MS/MS), represent a major advancement in CAH screening due to their superior analytical specificity. LC-MS/MS enables simultaneous quantification of multiple adrenal steroids and the calculation of diagnostic ratios—such as (17-OHP + Δ4-androstenedione)/cortisol—that markedly enhance discrimination between affected and unaffected infants. In some cohorts, this ratio has achieved a positive predictive value of 100% at optimized cutoffs, effectively eliminating false-positive results. Furthermore, LC-MS/MS second-tier testing has been shown to significantly improve overall screening performance and reduce unnecessary referrals, reinforcing its role as the preferred method for refining initial screen-positive results [30,31].

The progression from immunoassay to RIA and ultimately to LC-MS/MS reflects a broader shift toward more precise characterization of neonatal steroidogenesis. This evolution is particularly relevant when investigating whether newborn 17-OHP levels may serve as early indicators of disease severity in non-classical congenital adrenal hyperplasia (NCCAH). As screening programs adopt more specific methodologies, the neonatal steroid measurements available for research become increasingly reliable, strengthening the foundation for studies seeking to link early biochemical patterns with later clinical outcomes. Understanding how assay performance influences newborn 17-OHP values is therefore essential for interpreting their potential role as early markers of disease trajectory in NCCAH.

Delayed diagnosis may increase the risk of early pubertal progression, compromised adult height, infertility and psychosocial burden, underscoring the need for improved characterization of early predictors of disease expression [32,33,34,35,36,37,38,39,40,41,42,43,44,45].

The aim of the present study was to evaluate neonatal screening 17-OHP values and to provide a comprehensive description of the clinical, auxological, hormonal, and therapeutic characteristics of a pediatric cohort with genetically confirmed NCCAH. Particular emphasis was placed on the relationship between neonatal hormonal findings, biochemical severity at diagnosis, age at clinical onset, and glucocorticoid treatment requirements.

## 2. Materials and Methods

### 2.1. Study Design and Population

This retrospective observational study included pediatric patients diagnosed with NCCAH between January 2010 and December 2023 at a tertiary pediatric endocrinology center. Inclusion criteria were: (1) clinical suspicion of androgen excess, (2) biochemical evidence of 21-hydroxylase deficiency, (3) confirmation by ACTH stimulation test, and (4) genetic confirmation of CYP21A2 mutations consistent with NCCAH. Exclusion criteria were the lack of neonatal screening 17OHP levels or biochemical data at diagnosis or genetic data, and other conditions that may interfere with puberty onset.

### 2.2. Data Collection

The data were retrieved from medical records and included the following variables:

- Sex and age at clinical onset;
- Anthropometric parameters (height and weight SDS per gender and age);
- Clinical signs at presentation, including premature or early pubarche and premature or early gonadarche;
- Results of luteinizing hormone-releasing hormone (LHRH) stimulation test when performed;
- Neonatal screening 17-OHP levels;
- Basal and ACTH-stimulated 17-OHP levels at diagnosis;
- Basal and ACTH-stimulated cortisol levels at diagnosis;
- Bone age and degree of advancement relative to chronological age;
- Genetic findings;
- Hydrocortisone treatment and dosage;
- Hormonal assessment.

ACTH stimulation tests were performed using a standard high-dose protocol (250 µg synthetic ACTH, Synacthen^®^, Alfasigma, Bologne, Italy), administered intravenously as a slow one-minute bolus. Serum 17-OHP levels were measured at baseline and at 30 and 60 min after stimulation. Serum 17-OHP concentrations were determined by liquid chromatography–tandem mass spectrometry (LC-MS/MS) using an XEVO TQ-S micro system (Waters Corporation, Milford, MA, USA). Sample preparation and chromatographic separation followed the manufacturer’s instructions for the CE-IVD MassChrom^®^ 17-OHP assay (Chromsystems Instruments & Chemicals GmbH, Munich, Germany). Basal 17-OHP concentrations >2 ng/mL were considered suggestive of NCCAH, while post-stimulation levels >10 ng/mL were considered diagnostic. Serum cortisol levels were measured at baseline and at 30 and 60 min after stimulation and were determined by electrochemiluminescence immunoassay (Cobas module, Roche GmbH, Mannheim, Germany). Basal levels below 54 mcg/L and stimulated levels below 180 mcg/L were considered as diagnostic for adrenal insufficiency. Central precocious puberty was diagnosed based on a pubertal luteinizing hormone (LH) response to LHRH stimulation testing. Follicle-stimulating hormone (FSH) and Luteinizing Hormone (LH) were measured at baseline and at 20 and 40 min after stimulation with LHRH (Ferring GmbH, Kiel, Germany). LH and FSH concentrations were determined by electrochemiluminescence immunoassay (Cobas module, Roche GmbH, Mannheim, Germany). Stimulated LH and FSH levels >5 mIU/L were considered diagnostic for central precocious puberty.

CAH newborn screening was performed using the GSP Neonatal 17-OHP kit (Wallac Oy, Turku, Finland), which allows the quantitative determination of 17-hydroxyprogesterone from dried blood spot samples collected on Guthrie cards by heel prick at 48–72 h of life. All neonates with 17-OHP concentrations above 12 ng/mL among term infants were referred to the Pediatric Endocrinology Reference Center.

This retrospective observational study was conducted in accordance with the Declaration of Helsinki, and approved by The Interinstitutional Ethics Committee of A.O.U. Città della Salute e della Scienza di Torino—A.O. Ordine Mauriziano—A.S.L. “Città di Torino” (protocol code 1289/A, 17 April 2021)

### 2.3. Statistical Analysis

Continuous variables are reported as mean ± standard error of the mean (SEM). Correlations between hormonal parameters, age at clinical onset, and hydrocortisone dosage were assessed using appropriate correlation tests according to data distribution. Statistical analysis was performed using GraphPad Prism version 9 (GraphPad Software, La Jolla, CA, USA). Associations between variables were evaluated using Student’s *t* test, and a *p* value < 0.05 was considered statistically significant. 

## 3. Results

### 3.1. Study Population and Clinical Presentation

Thirty-five pediatric patients with genetically confirmed NCCAH were included in the study, comprising five males (14.3%) and thirty females (85.7%), as shown in Table 1. The mean age at clinical onset was 7.52 ± 0.36 years for females and 6.25 ± 0.29 years for males.

At diagnosis, auxological assessment revealed a mean height SDS of 1.85 ± 0.34 and a mean weight SDS of 1.75 ± 0.29, indicating accelerated growth compared with age- and sex-matched reference populations, as also confirmed by the growth velocity mean SDS 1.97 ± 0.54.

Premature pubarche was the most common presenting sign, observed in 33 out of 35 patients (94.3%). Gonadarche was present in 15 patients (42.9%). Eleven patients (31.4%) were diagnosed with central precocious puberty, confirmed by a pubertal LH response to LHRH stimulation testing.

### 3.2. Neonatal Screening Data

Neonatal screening 17-OHP values were available for all patients. The mean screening 17-OHP concentration was 4.53 ± 0.7 ng/mL. Two patients (5.7%) had a value below 2 ng/mL, 31 patients (88.6%) had values between 2 and 12 ng/mL, and only two patients (5.7%) exceeded the referral cut-off value of 12 ng/mL, thus triggering neonatal recall.

### 3.3. Hormonal Findings at Diagnosis

At diagnosis, the mean basal serum 17-OHP concentration was 15.1 ± 3.35 ng/mL. All patients underwent high-dose ACTH stimulation testing, which revealed a mean stimulated 17-OHP value of 55.2 ± 11.3 ng/mL. Bone age advancement was observed in all patients, with a mean difference between bone age and chronological age of 2.15 ± 0.34 years. Normal basal cortisol levels were observed in all patients whereas ACTH-stimulated cortisol levels were below 180 mcg/L in 11/35 patients (31.4%).

### 3.4. Genetic Analysis

Homozygous p.Val282Leu (c.844G>T) mutations in the *CYP21A2* gene were identified in 19/35 patients (54.3%), while the remaining individuals carried compound heterozygous mutations (Table 2). Compared with the other patients, those with homozygous p.Val282Leu mutations were younger at diagnosis (6.45 ± 0.28 vs. 8.1 ± 0.49 years; *p* = 0.005) and had higher basal (22.2 ± 5.67 vs. 6.63 ± 0.92 ng/mL; *p* = 0.01) and stimulated (69.8 ± 18.46 vs. 27.6 ± 3.39 ng/mL; *p* = 0.04) 17OHP levels. Moreover, patients with homozygous p.Val282Leu mutations showed a higher rate of ACTH-stimulated cortisol levels below 18 µg/dL compared with the other patients (52.6% vs. 6.25%; *p* = 0.003).

### 3.5. Treatment and Correlation Analyses

Thirty-three patients (94.3%) initiated hydrocortisone therapy, with a mean dose of 11.9 ± 0.51 mg/m^2^/day. Age at clinical onset showed a significant inverse correlation with basal 17-OHP levels (*p* = 0.01) and with ACTH-stimulated 17-OHP levels (*p* = 0.03), indicating that patients with more pronounced biochemical abnormalities presented at a younger age (Table 3). Furthermore, hydrocortisone dosage was positively correlated with both basal and stimulated 17-OHP concentrations at diagnosis (*p* = 0.01 and *p* = 0.02, respectively), reflecting a relationship between biochemical severity and treatment requirements.

## 4. Discussion

In this study, we provide a comprehensive clinical, hormonal, and neonatal screening characterization of a pediatric cohort with genetically confirmed NCCAH. Our findings confirm the limited sensitivity of neonatal screening for NCCAH, highlight the high prevalence of androgen-related clinical manifestations during childhood, and demonstrate significant associations between biochemical severity, age at onset, and glucocorticoid treatment requirements [17,18,19,20,21].

Consistent with previous reports, most patients in our cohort showed neonatal 17-hydroxyprogesterone (17-OHP) values that were mildly elevated but did not exceed the screening recall threshold. Only two patients were identified by neonatal screening, despite nearly 90% of patients presenting values above the lower limit of normal. These findings align with large population-based studies indicating that neonatal screening programs, although highly effective in detecting classical salt-wasting CAH, fail to identify most cases of NCCAH due to insufficiently elevated 17-OHP concentrations at birth. This reflects the residual enzymatic activity typically present in NCCAH, which generally prevents neonatal adrenal insufficiency while permitting progressive androgen excess over time. However, adrenal insufficiency should be ruled out in all cases of NCCAH. The rate of adrenal insufficiency in our cohort was consistent with previously published papers [9,10,11,12,13,14].

Importantly, our data support the concept of a biochemical continuum rather than a strict dichotomy between classical and non-classical CAH. Neonatal 17-OHP concentrations in patients with NCCAH were frequently above the population mean, suggesting that subtle hormonal alterations are already present at birth. However, the individual clinical relevance of these mildly elevated values remains difficult to interpret, given the intentionally high screening cut-offs designed to limit false-positive results, particularly in preterm or stressed neonates. At present, lowering screening thresholds would likely result in an unacceptable increase in false-positive cases, limiting the feasibility of neonatal detection of NCCAH within population-based screening programs [17,18,19,20,21].

From a clinical perspective, premature pubarche emerged as the predominant presenting sign, occurring in more than 90% of patients, in line with previous pediatric series [9,10,11,12,13,14]. Accelerated linear growth and advanced bone age were universal findings, underscoring the impact of prolonged androgen exposure on skeletal maturation. Notably, nearly one-third of patients developed central precocious puberty, a prevalence at the upper range of that reported in the literature. This observation supports the hypothesis that chronic peripheral androgen excess may facilitate early activation of the hypothalamic–pituitary–gonadal axis, further amplifying the risk of compromised adult height.

These observations have direct clinical implications. A normal or non-recalled neonatal screening result should not be considered sufficient to exclude NCCAH, and clinicians should maintain a high index of suspicion in children presenting with premature pubarche, accelerated growth, and especially advanced bone age [14]. In such cases, early hormonal evaluation, including basal and ACTH-stimulated 17-OHP measurement, may facilitate timely diagnosis and allow for individualized treatment decisions, potentially reducing the risk of early pubertal progression and long-term growth impairment.

A key finding of our study is the inverse correlation between age at clinical onset and both basal and ACTH-stimulated 17-OHP levels, suggesting that biochemical severity is a major determinant of phenotypic expression in NCCAH and correlates with genotype. This finding is consistent with genotype–phenotype correlation studies demonstrating that mutations associated with lower residual 21-hydroxylase activity lead to earlier and more pronounced clinical manifestations [4,5,6,7,8].

Furthermore, the observed positive correlation between hydrocortisone dosage and 17-OHP concentrations at diagnosis supports an individualized therapeutic approach. Current recommendations emphasize that glucocorticoid treatment in NCCAH should be reserved for symptomatic patients or those at risk of compromised growth, to avoid overtreatment and potential long-term adverse effects [22]. Our findings suggest that baseline hormonal evaluation may provide useful guidance in tailoring treatment intensity. Higher than expected mean doses were observed in our cohort as initial doses were higher, especially in patients showing sub-optimal ACTH-stimulated cortisol levels, and were then tapered during follow-up.

The major strength of this study is the inclusion of a well-characterized pediatric cohort with genetically confirmed NCCAH, which minimizes diagnostic uncertainty and distinguishes this series from studies relying solely on biochemical criteria. In the Piedmont region of Italy, neonatal screening for 17OHP is routinely performed in all newborns. During the study period, nearly 300,000 infants underwent screening for 17OHP levels. The availability of neonatal screening 17-OHP values for all patients allowed a direct evaluation of early hormonal patterns in relation to later clinical expression. Unfortunately, most subjects exhibited 17OHP levels above 2 ng/mL but below 12 ng/mL, the established screening threshold for recall. Within this “grey zone,” many newborns without non-classical congenital adrenal hyperplasia (NCCAH) may present with similar values, making identification of NCCAH through neonatal screening particularly challenging. Moreover, 17OHP concentrations may be transiently elevated during the neonatal period due to factors such as prematurity, perinatal stress, or illness, further reducing the specificity of screening. Nevertheless, when NCCAH is clinically suspected, neonatal screening levels of 17OHP—if available—should be included in the initial assessment. In addition, the comprehensive assessment of auxological, hormonal, pubertal, and therapeutic parameters enabled an integrated analysis of disease severity and treatment needs in a real-world clinical setting.

Several limitations should be acknowledged. The retrospective design and relatively small sample size may limit the generalizability of the findings. Moreover, longitudinal outcome data, including final height and metabolic parameters, were not available. Variability in neonatal screening assays and cut-off values across regions may also influence the applicability of these results to different screening programs. Nonetheless, the genetic confirmation of all diagnoses and the comprehensive assessment of neonatal, clinical, and hormonal data represent notable strengths of this study.

## 5. Conclusions

Neonatal screening for congenital adrenal hyperplasia is highly effective in identifying classical forms of the disease but fails to detect most cases of non-classical congenital adrenal hyperplasia. In our cohort, neonatal 17-OHP values were frequently mildly elevated but below recall thresholds, leading to delayed diagnosis until the appearance of clinical signs in childhood.

Higher basal and ACTH-stimulated 17-OHP concentrations were associated with earlier clinical onset, advanced bone age, increased risk of central precocious puberty, and greater glucocorticoid treatment requirements. These findings underscore the clinical relevance of biochemical severity at NCCAH diagnosis in shaping disease expression and therapeutic needs.

In the absence of reliable neonatal diagnostic markers for NCCAH, heightened clinical awareness and careful hormonal evaluation remain essential in children presenting with early signs of androgen excess, regardless of neonatal screening results. Future studies should focus on identifying early predictors of disease severity and on optimizing long-term management strategies to improve growth and pubertal outcomes in this population.

## Figures and Tables

**Table 1:** Clinical, Biochemical and Treatment Characteristics of the Study Population (N = 35).

**Demographic** **characteristics (n = 35)**	Female, n (%)	30 (85.7%)
	Male, n (%)	5 (14.3%)
	Mean age at clinical onset for females, years (mean ± SEM)	7.52 ± 0.36
	Mean age at clinical onset for males, years (mean ± SEM)	6.25 ± 0.29
**Auxological** **data at diagnosis**	Height SDS (mean ± SEM)	1.85 ± 0.34
	Weight SDS (mean ± SEM)	1.75 ± 0.29
	Growth velocity SDS (mean ± SEM)	1.97 ± 0.54
	Bone age advancement, years (BA − CA, mean ± SEM)	2.15 ± 0.34
**Clinical presentation**	Premature pubarche, n (%)	33 (94.3%)
	Premature gonadarche, n (%)	15 (42.9%)
	Central precocious puberty, n (%)	11 (31.4%)
**Neonatal screening** **(17-OHP)**	Mean 17-OHP, ng/mL (mean ± SEM)	4.53 ± 0.7
	<2 ng/mL, n (%)	2 (5.7%)
	2–12 ng/mL, n (%)	31 (88.6%)
	>12 ng/mL (recall), n (%)	2 (5.7%)
**Hormonal findings** **at diagnosis**	Basal 17-OHP, ng/mL (mean ± SEM)	15.1 ± 3.35
	ACTH-stimulated 17-OHP, ng/mL (mean ± SEM)	55.2 ± 11.3
	Basal Cortisol, mcg/L (mean ± SEM)	102.4 ± 25.4
	ACTH-stimulated Cortisol mcg/L (mean ± SEM)	211 ± 45.4
**Treatment**	Hydrocortisone therapy initiated, n (%)	33 (94.3%)
	Hydrocortisone dose, mg/m^2^/day (mean ± SEM)	11.9 ± 0.51

**Table 2:** Genetic analysis of NCCAH patients.

Genetic findings	p.Val282Leu c.844G>T (homozygous)	19 (54.2%)
	c.518T>A (p.Ile173Asn), c.1360C>T (p.Pro454Ser)	2 (5.6%)
	p.Val282Leu c.844G>T, c.1360C>T (p.Pro454Ser)	2 (5.6%)
	c292-13 A/C>G (Introne 2), p.Val282Leu c.844G>T	2 (5.6%)
	p.Val282Leu c.844G>T, c.955C>T (p.Gln319*)	1 (2.9%)
	pPro31Leu/pPro 483 Ser	1 (2.9%)
	p.Val282Leu c.844G>T, p.Trp398Ser	1 (2.9%)
	pVal281Leu/pPro30Leu	1 (2.9%)
	p.Val282Leu c.844G>T, c.923_924insT (p.Leu308PhefsTer6)	1 (2.9%)
	c289-13 A/C>G (Introne 2), p Ile 172 Asn	1 (2.9%)
	p.Arg357Trp/p.Val282Leu c.844G>T	1 (2.9%)
	c.-126C>G, p.Val282Leu c.844G>T	1 (2.9%)
	CYP21A2/pseudogene fusion, c.841G>T (p. Val281Leu)	1 (2.9%)
	Q318X (p.Gln318X), V281L (p.Val282Leu)	1 (2.9%)

**Table 3:** Age at onset and hydrocortisone dose correlation with basal and ACTH-stimulated 17-OHP.

Correlation analyses	Age at onset vs. basal 17-OHP	Inverse correlation (*p* = 0.01)
	Age at onset vs. stimulated 17-OHP	Inverse correlation (*p* = 0.03)
	Hydrocortisone dose vs. basal 17-OHP	Positive correlation (*p* = 0.01)
	Hydrocortisone dose vs. stimulated 17-OHP	Positive correlation (*p* = 0.02)

## Data Availability

Some or all datasets generated during and/or analyzed during the current study are not publicly available but are available from the corresponding author on reasonable request.

## Abbreviations

The following abbreviations are used in this manuscript:

NCCAH	Non-classical congenital adrenal hyperplasia
17OHP	17-hydroxyprogesterone
CAH	Congenital adrenal hyperplasia
ACTH	Adrenocorticotropic Hormone
CPP	Central precocious puberty
LHRH	Luteinizing Hormone-Releasing Hormone
RIA	Radioimmunoassay
LM-MS/MS	Liquid chromatography–tandem mass spectrometry
LH	Luteinizing Hormone
FSH	Follicle-stimulating Hormone
